# Using COM-B model in identifying factors influencing exercise adherence in pregnant women with gestational diabetes mellitus in China: a qualitative study

**DOI:** 10.3389/fpubh.2025.1736624

**Published:** 2026-01-22

**Authors:** Cai-Li Jia, Li-Jun Wang, Li-Hong Li, Ya-Juan Lu, Yue Yang

**Affiliations:** 1 Department of Gynecology and Obstetrics, Civil Aviation General Hospital, Beijing, China; 2 Nursing Department, Civil Aviation General Hospital, Beijing, China

**Keywords:** China, COM-B model, exercise adherence, GDM, influencing factors, qualitative study

## Abstract

**Background:**

Gestational Diabetes Mellitus (GDM) is a common complication of pregnancy and that uncontrolled GDM increases the risk of poor maternal and neonatal health outcomes.

**Objective:**

While regular exercise benefits GDM pregnant women, exercise adherence remains suboptimal; thus, this study aimed to identify the factors influencing exercise adherence among pregnant women with gestational diabetes in order to inform the development of tailored intervention to improve physical activity adherence and thereby promote more GDM pregnant women to achieve the recommended physical activity level.

**Methods:**

The descriptive qualitative study was conducted in the obstetric clinic of tertiary hospital in Beijing, China. A total of 16 pregnant women with GDM were recruited as participants via purposive sampling, and face-to-face semi-structured interviews were adopted to collect data. All the interviews were audio-recorded and timely transcribed. The directed content analysis was used to analyze data and the themes generated were mapped onto the Capability, Opportunity, Motivation and Behavior model components.

**Results:**

Sixteen women from a broad spectrum of socio-demographic backgrounds participated in this study. Three main themes and nine sub-themes were identified as shown below: Capability-related factors included: (1) absence of established exercise habits and exercise foundation, (2) physical discomfort with limited coping strategies, and (3) insufficient knowledge and skills regarding exercise during pregnancy. Opportunity-related factors comprised: (1) inadequate informational support and individualized guidance from healthcare professionals, (2) strong family support, and (3) insufficient peer support and community-based support. Motivation-related factors encompassed: (1) heightened risk perception of GDM promoting exercise intention, (2) recognition of exercise-related benefits enhancing motivation, and (3) negative emotions stemming from uncertainty about exercise safety.

**Conclusion:**

Our study found that limited engagement in pre-pregnancy and early pregnancy physical activity, challenges of physical discomfort, insufficient knowledge and skills regarding exercise during pregnancy, insufficient professional guidance, limited peer support and community-based support, and negative emotions related to uncertain exercise safety during pregnancy were notable barriers to compliance with exercise recommendations in women with GDM. This has clinical implications for healthcare professionals to design context-specific interventions to address these barriers, thereby improving exercise compliance and physical activity levels among women with GDM.

## 1 Introduction

Gestational diabetes mellitus (GDM) is a prevalent metabolic complication during pregnancy, with a range of adverse short-term and long-term health impacts on both mothers and offspring. Hyperglycemia in pregnancy affects an estimated one in six pregnancies globally, corresponding to 21 million women annually (1). A recent study demonstrated the prevalence of GDM in China was 15.6% (95%CI 14.9–16.2) (2), and with the implementation of revised fertility policies and a rising maternal age, the incidence of GDM has been increasing annually (3). The increasing occurrence rate of GDM gives rise to a tremendous burden on both the individuals and the country in China (4, 5). Therefore, it is of great significance to focus on the prevention and intervention of GDM to reduce its adverse effects on maternal and child health.

Physical activity (PA) is one of the important measures for the treatment and management of GDM, which has been widely recognized. Intensive studies have demonstrated that exercise was beneficial for glucose metabolism, pregnancy outcome, and the reduction of medicine use (6–9). Guidelines worldwide recommend that healthy pregnant women should undertake regular physical activity throughout pregnancy and achieve at least 150 min of exercise at moderate intensity per week (10). In China, GDM pregnant women without exercise contraindications are recommended to maintain appropriate exercise, and at least 5 days per week, at least 30 min per day, or at least 150 min of moderate intensity exercise per week (11). However, evidence has indicated that physical activity levels among pregnant women generally fall below these recommendations and tend to decline further during the third trimester (12, 13). In China, only 21.0% of pregnant women met the recommended PA level according to a systematic review and meta-analysis of 12 cross-sectional studies and 11,323 Chinese pregnant women (14). Furthermore, physical activity levels among GDM pregnant women are generally inadequate (15, 16). Therefore, identifying the factors influencing exercise compliance in GDM pregnant women is the first step to develop effective strategies to improve their physical activity levels. Numerous studies in China have identified factors influencing physical activity levels among pregnant women, including employment status, pre-pregnancy exercise habits, pregnancy trimester, pre-pregnancy body mass index (BMI), and physical discomfort during pregnancy (14). However, most of these factors were reported from cross-sectional studies, there are few qualitative studies on the influencing factors of exercise compliance in women with GDM. Research also suggested the use of theoretical frameworks can be more effective when understand behavior change, with the Capability, Opportunity, Motivation and Behavior model (COM-B model) being a recommended approach (17).

The COM-B Model is a theoretical framework for behavior change that provides a structure for analyzing and understanding the factors influencing an individual’s behavior. The acronym COM-B stands for three core components: Capability, Opportunity, and Motivation. These three components interact with one another to collectively determine an individual’s behavior (17). Capability refers to the psychological and physical abilities an individual needs to perform a behavior; Opportunity encompasses external environmental factors such as social and physical environments; Motivation includes automatic psychological processes (e.g., habits and emotions) and reflective processes (e.g., beliefs and goals) that activate or inhibit behavior (17). The COM-B Model incorporates context in understanding behavior and developing behavior change intervention, while providing a systematic method for analyzing the target behavior and characterizing interventions based on the behavior diagnosis (17). Therefore, the COM-B model has been applied to many clinical problems such as disease prevention, health promotion, and self-management, but has not yet been applied to the exercise compliance of women with GDM. The aim of this study was to explore lived experience of physical activity of pregnant women with GDM and identify the influencing factors of their exercise adherence guided by the COM-B model, with a view to informing the development of a tailored intervention aimed at improving physical activity compliance in this population.

## 2 Methods

### 2.1 Study design

We adopted the purposive sampling approach with maximal variation including diverse ages, parity, educational level, occupations, prior history of GDM, family history of T2DM, insulin treatment status (yes/no), and oral glucose tolerance test (OGTT) results at GDM diagnosis. Semi-structured face-to-face interviews were conducted with eligible participants in Beijing, China. Directed content analysis method was used to analyze qualitative data. This study received review and approval from the Ethics Committee of Civil Aviation General Hospital (2025-Y-D-021).

### 2.2 Participants

The study was carried out between June 2024 and October 2024. Eligible participants who received regular antenatal care were recruited from the obstetrics clinic of a tertiary general hospital in Beijing, China.

#### 2.2.1 Inclusion criteria of participants

(a) Diagnosis of GDM confirmed by a 75 g oral glucose tolerance test (OGTT) administered between 24 and 28 weeks of gestation (18); (b) Age ≥ 18 years; (c) Singleton pregnancy; d. Adequate language proficiency and communication abilities; (e) Provision of informed consent and voluntary participation in the study.

#### 2.2.2 Exclusion criteria of participants

(a). Multiple pregnancy; (b) Presence of severe pregnancy-related complications or comorbidities; (c) Presence of medical contraindications to physical activity (18).

We checked the prenatal examination data of pregnant women diagnosed with GDM, and screened eligible participants. Then potential participants for inclusion and exclusion criteria were contacted by phone. And explained to them the purpose, significance, and process of this study, and ask if they are willing to participate in this study. If agreed, determine a mutually convenient time. The sample size required was determined when saturation of themes was achieved (19).

The final sample size was determined by achieving saturation of themes during the interviews, with two additional participants interviewed thereafter to confirm data saturation and ensure the comprehensiveness of the findings. A total of 16 women with GDM were interviewed for the study and assigned identification numbers 1–16 to maintain confidentiality. The demographic and clinical characteristics of the participants are summarized in Table 1.

**Table 1:** Socio-demographic and clinical characteristics of study participants (n = 16).

Variables	mean ± SD/*n*(%)
Age, years, mean ± SD	31.50 ± 4.47
Gestational age, weeks, mean ± SD	35.97 ± 1.64
Level of Education	
Master’s degree, *n* (%)	4 (25.0%)
Bachelor’s degree, *n* (%)	7 (43.75%)
College degree, *n* (%)	3 (18.75%)
High school, *n* (%)	1 (6.25%)
Middle school, *n* (%)	1 (6.25%)
Employment status^*^	
Employed professional, *n* (%)	12 (75.0%)
Self-employed, *n* (%)	2 (12.5%)
Not employed, *n* (%)	2 (12.5%)
Parity^**^	
Primipara, *n* (%)	10 (62.5%)
Multipara, *n* (%)	6 (37.5%)
Pre-pregnancy Weight^***^	
Obesity, *n* (%)	4 (25.0%)
Overweight, *n* (%)	2 (12.5%)
Normal weight, *n* (%)	9 (56.25%)
Underweight, *n* (%)	1 (6.25%)
Prior History of GDM^****^	
Yes, *n* (%)	3 (18.75%)
No, *n* (%)	13 (81.25%)
Family history of T2DM^*****^	
Yes, *n* (%)	1 (6.25%)
No, *n* (%)	15 (93.75%)
Insulin treatment	
Yes, *n* (%)	8 (50%)
No, *n* (%)	8 (50%)
Oral Glucose Tolerance Test (OGTT) results^******^	
Fasting plasma glucose ≥5.1 mmol/L, *n* (%)	5 (31.25%)
1-h plasma glucose ≥10.0 mmol/L, *n* (%)	9 (56.25%)
2-h plasma glucose ≥8.5 mmol/L, *n* (%)	11 (68.75%)

* Professional employment included clerk, civil servant, nurse, doctor and kindergarten teacher.

** There are 6 multiparous women, among whom 3 will give birth to her second child, 2 will give birth to her third child, and 1 will give birth to her fourth child.

*** Pre-pregnancy weight is classified based on pre-pregnancy body mass index (BMI), which was categorized as underweight (BMI < 18.5 kg/m^2^), normal weight (18.5 kg/m^2^ ≤ BMI < 24.0 kg/m^2^), overweight (24.0 kg/m^2^ ≤ BMI < 28.0 kg/m^2^) and obesity (BMI ≥ 28.0 kg/m^2^).

**** GDM: Gestational Diabetes Mellitus.

***** T2DM: Type 2 Diabetes Mellitus.

****** GDM is diagnosed via a 75 g OGTT if any of the following blood glucose thresholds are met or exceeded: fasting blood glucose ≥ 5.1 mmol/L, blood glucose at 1-h after glucose ingestion ≥ 10.0 mmol/L, and blood glucose at 2-h after glucose ingestion ≥ 8.5 mmol/L.

### 2.3 Development of the interview outline

Guided by the COM-B model, design a preliminary interview outline through literature review, group discussions, and expert consultation. Following two pilot interviews with two GDM pregnant women, the formal interview outline has been revised and confirmed as follows:

1. Could you describe your exercise habits prior to pregnancy, including types of exercise, frequency, duration, intensity?
2. What changes, if any, have occurred in your exercise routine since becoming pregnant?
3. What factors or considerations contributed to these changes?
4. What is your opinion on exercise for pregnant women with GDM?
5. To what extent do you adhere to your exercise plan? What aspects were followed, and what aspects were not?
6. What factors help you follow the exercise plans?
7. What factors affect your following the exercise plans?
8. What methods do you use to deal with these challenges?
9. What forms of support or assistance have you received while following the exercise plans?
10. What are your thoughts and expectations regarding the current exercise plan?

### 2.4 Data collection

Data collection was conducted through face-to-face semi-structured interviews. The interviewer had received formal training in qualitative research methodologies. Interviews were carried out as schedule in a quiet and private outpatient clinic education room to ensure a comfortable and confidential environment. Before initiating the interview, the interviewer introduced their name and identity to the interviewee, explained to them why we were interested in the study and why we hoped to learn about the interviewee’s views and experiences through this interview, informed them of the research process and schedule, ensured that the interviewee understood their required time and level of participation. The participants were informed that the principles of confidentiality and privacy protection would be followed and the reason why each interview was audio-recorded. In addition, they were assured that they were free to withdraw at any time during the interview without any consequences. The above mentioned were all aimed at establishing a relationship of mutual trust and rapport. The interviewer obtained written informed consent from interviewees to start the recording and began the semi-structured interview.

The interview process allowed for flexibility in the phrasing and sequencing of questions, which was adjusted in response to the context and comfort level of the participant. Care was taken to avoid the use of leading or suggestive language. Participants were encouraged to express their experiences and perspectives freely and in detail. The interviewer actively listened, posed relevant follow-up questions, and documented non-verbal cues such as gestures, facial expressions, and vocal tone. Clarification was provided as needed when participants raised questions. After each interview, the interviewee filled out a survey questionnaire on socio-demographic data.

### 2.5 Data analysis

Data collection and data analysis were conducted simultaneously in this study. Following each interview, the audio recordings were reviewed multiple times and transcribed verbatim within 24 h. During the transcription process, relevant non-verbal elements such as emotional tone, vocal inflections, and gestures were documented to preserve contextual richness. Data were analyzed using directed content analysis (20, 21). The steps of the analysis included the followings (22–24): (1) The transcripts that reflected the influencing factors of exercise compliance of pregnant women with GDM were used as the minimum analysis units. (2) The initial data including the recordings, the transcripts and field notes were reviewed and read repeatedly to get an overall impression of the data. (3) The COM-B model was used as a framework to categorize the unit of analysis. (4) Content encoding and classification, annotating important ideas and concepts in the data, and grouping similar codes into corresponding categories to form themes and sub themes: (5) Interpretation and analysis of the results, establishing a connection between data and results, and identifying corresponding examples of excerpts from the data.

## 3 Results

### 3.1 Study participant characteristics

Table 1 presented the socio-demographic characteristics of study participants. A total of 16 participants were interviewed. The mean age of them was (31.50 ± 4.47) years and the mean gestational age was (35.97 ± 1.64) weeks. Among the participants, 68.75% hold a bachelor’s or master’s degree and 62.5% of them were primipara. The majority is professional employment, accounting for 75.0%, while self-employed and unemployed individuals accounted for 25.0%. 56.25% of them were normal body mass index (BMI), 25.0% were obesity, 12.5% were overweight while 6.25% were underweight. In total, 81.25% of them did not have a history of GDM and 93.75% did not have family history of type 2 diabetes (T2DM). Whether to receive insulin treatment accounted for 50% each. In the oral glucose tolerance test (OGTT) results, 5 out of 16 participants (31.25%) had a fasting blood glucose level exceeding the diagnostic threshold, and 9 participants (56.25%) and 11 participants (68.75%) presented with elevated blood glucose levels at 1 h and 2 h after glucose ingestion, respectively. No participants withdrew from the interviews midway or declined to respond to any questions. The duration of each interview ranged from 20 to 40 min.

Three themes and nine sub-themes were generated from our analysis and mapped them onto the COM-B model as shown below: (1) capability-related factors included absence of established exercise habits and exercise foundational, physical discomfort and limited coping strategies, and insufficient knowledge and skills regarding pregnant exercise; (2) opportunity-related factors comprised strong family support, inadequate informational support and individualized guidance from healthcare professionals, and insufficient peer and community-based support; (3)motivation-related factors encompassed heightened risk perception of GDM promoting exercise intention, recognition of exercise-related benefits enhancing motivation, and negative emotional experiences stemming from uncertainty about exercise safety.

### 3.2 Capability factors

#### 3.2.1 Lack of exercise habits and foundation

Most participants reported that the low involvement of physical activity before being diagnosed with GDM as a result of absence of exercise habits and foundation. Some participants further substantiated that despite being aware of the benefits of exercise during pregnancy, the development of exercise habits is influenced by some reasons such as busy work schedules, work fatigue, or traditional belief that conception requires more rest and taking more rest during early pregnancy can prevent miscarriage. As noted by participants below:

“I know that exercise is beneficial, but I hardly do it mainly because of my busy work schedule” (No 1).

“I hardly exercised in my early pregnancy. Since I had two miscarriages before, I spent the first few months of this pregnancy staying at home and resting in bed” (No 6).

“Going to work every day is already exhausting. I commute from the east fifth ring road to the north fifth ring road, switching subway lines, and on top of my daily workload, I did not make an effort to exercise” (No 7).

“Before being diagnosed with diabetes, I had a particularly bad habit. Just because I became pregnant, I became very dependent. I lay down immediately after meals and felt very tired. But in fact, these things can be overcome” (No 12).

#### 3.2.2 Physical discomfort and limited coping abilities

Several participants reported that physical discomfort associated with pregnancy significantly limited their ability to engage in physical activity. As gestation progressed, symptoms such as fatigue, lower extremity edema, tightness in the lower abdomen and lower back pain became increasingly prevalent. Moreover, As exemplified by participants below, due to limited coping strategies for managing these discomforts, resting was frequently adopted as a primary means of symptom relief, greatly restricting physical activity and making it difficult to incorporate exercise into their life routines.

“My morning sickness was quite severe; I felt lethargic and always wanted to lie down” (No 2).

“By the end of the day, I am exhausted and have back pain. When I get home from work, I do not want to move at all” (No 3).

“I believe that exercise is extremely beneficial and necessary. However, as the pregnancy advances, walking causes my abdomen to tighten and harden, and I experience pubic pain. My physical condition does not allow me to walk much, but if I could, I would like to move around more, which would be beneficial during the delivery” (No 11).

#### 3.2.3 Lack of knowledge and skills regarding exercise during pregnancy

Majority of these participants reported that low participation in physical activity was as a result of limited knowledge or skills about pregnant physical activity and limited awareness on exercise methods and intensity. As shown below, these opinions were common.

“Before I was diagnosed with high blood glucose, I thought everything was fine, so I did not exercise at all” (No 6).

“There was a period when the doctor told me to walk for half an hour, but I insisted on walking for an hour. However, later I felt extremely tired, and there were several times when I could barely make it back halfway through the walk. Eventually, I gave up” (No 11).

In addition to the lack of pregnant exercise knowledge, several respondents opined that it was not necessary for pregnant women to take physical activity. As echoed by participant below, the importance of exercise during pregnancy seemed unclear.

“Before taking OGTT, I thought I wasn’t overweight, so I did not exercise much, believing there is no need for me to exercise” (No 13).

### 3.3 Opportunity factors

#### 3.3.1 Strong family support

Most participants described receiving various forms of support from spouses, family members, which positively influenced their motivation and adherence to physical activity. As substantiated by some participants below, family supports especially emotional support in the form of companionship and encouragement from family members, was frequently highlighted as a key facilitator of exercise engagement.

“Now, exercising has become a task for both of us. Every day when it’s time, my husband pulls me downstairs to go for a walk” (No 6).

“Recently, my younger sister came home, and she keeps reminding me to go out for a stroll. Having someone accompany me to exercise makes it much easier” (No 8).

“Because (exercise) requires a bit of an increase in pace, my husband joins me. It’s easy to get tired when walking alone but walking while chatting together makes it easier to persist” (No 12).

#### 3.3.2 Insufficient informational support and personalized guidance from healthcare providers

Some participants reported that the exercise guidance received from healthcare providers was perceived as insufficiently specific. The information was often limited to general oral advice and lack of practical detail and individualized applicability. As exemplified by participants below, the lack of tailored instruction led to poor compliance and more professional and personalized supports were needed.

“I found some maternal exercises on Douyin (TikTok), and I saw pregnant women still doing them at 38 or 39 gestational weeks, but I did not dare to follow along. If our doctors or nurses could guide us through the recommended exercises, I would feel much more reassured” (No 1).

“In summer, it’s really challenging to go out for a walk, especially after lunch when it’s the hottest time of the day. If there were a scientific exercise plan that could be done indoors, I think it would be perfect” (No 11).

Furthermore, several participants exhibited the guidance from healthcare providers was perceived as relatively conservative. As noted by participants below.

“The doctor told me to walk more at home, but they were quite vague about how fast and how long I should walk. I think doctors might be somewhat cautious in this regard” (No 13).

#### 3.3.3 Limited peer support and community-based support

Most participants described receiving various forms of support from family members. Conversely, most participants do not receive support from the peers and the community, only one participant reported she received support from the community, as narrated by the participant below, the social support from community healthcare providers was also the facilitator of exercise engagement.

“When registering at the community health center, the community doctor added us to a WeChat group where many pregnant women shared insights and experiences about exercise. Listening to those good suggestions is also very helpful. It would be great if you could also establish such a group” (No 6).

### 3.4 Motivation factors

#### 3.4.1 Risk perception of disease sparks exercise motivation

Some participants expressed a clear understanding of the health risks associated with GDM, particularly regarding potential adverse outcomes for the fetus and the risk awareness served as a strong internal motivator, prompting the voluntary engagement in physical activity.

“I started exercising primarily for the sake of my child. The doctor told me about potential risks such as macrosomia and neonatal hypoglycemia and hearing all this made me gradually commit to (exercising)” (No 4).

“The main reason I persist in exercising is to avoid any long-term effects to my baby due to GDM” (No 9).

“After confirming that I had GDM, the doctor mentioned several potential hazards, including impacts on the baby’s development and possible effects on heart health. Facing these risks, you realize that no matter how hard or tiring it might be, ensuring a healthy baby is worth it” (No 12).

#### 3.4.2 Perceived benefits of exercise increase motivation for physical activity

Some participants reported experiencing notable benefits following engagement in regular physical activity. As outlined by participants below, the perceived benefits included improved blood sugar level, relief from constipation, and reduction in negative emotional states.

“Before, when I did not like walking, it felt very strenuous. Now, I feel much more agile, and my constipation has improved significantly” (No 6).

“Sometimes, when I accidentally eat a little more than I should, if I do not exercise, my blood glucose levels will rise. However, after exercising, not only does it ease my psychological concerns, but it genuinely shows an effect on the numerical values” (No 12).

#### 3.4.3 Negative emotional experiences due to uncertainty about exercise safety

Some study participants reported experiencing psychological distress related to uncertainties surrounding the safety of exercise during pregnancy. As mentioned by some participants below, concerns about potential adverse consequences such as umbilical cord entanglement, premature rupture of membranes, and increased physical discomfort have led to anxiety and hesitation regarding physical activity.

“When exercising, I’m mainly worried about the impact on the baby. When my body moves, I’m afraid of causing a nuchal cord or rupturing the amniotic sac. Thus, I hardly exercise anymore” (No 1).

“The doctor advised me to exercise more, so I just take a walk after meals. I always feel that more intense activities are unsafe” (No 2).

“If I exercise, it’s mostly just a casual stroll after meals. I constantly worry that excessive movement might affect the fetus, and I’m also concerned that an elevated heart rate might have some adverse impact” (No 9).

## 4 Discussion

This research paper sought to identify the influencing factors of exercise compliance among pregnant women with GDM in Beijing located in the northern China. Our study findings illustrate that there are multiple factors that influence women’s perceptions and behavior towards exercise involvement during pregnancy. These factors were mapped onto the COM-B model and reported as follows: absence of established exercise habits and exercise foundation, physical discomfort and limited coping strategies, insufficient knowledge and skills regarding pregnant exercise (capability-related factors), inadequate informational support and individualized guidance from healthcare professionals, strong family support, insufficient peer support and community-based support (Opportunity-related factors) and heightened risk perception of GDM promoting exercise intention, recognition of exercise-related benefits enhancing motivation, negative emotions stemming from uncertainty about exercise safety (Motivation-related factors).

### 4.1 Capability-related factors

The COM-B component of capability was highly salient in this study, which restricts women involvement in PA through limited exercise habit and knowledge on pregnant PA. Although numerous studies have shown that engaging in regular physical activity pre and during pregnancy can significantly reduce the risk of developing GDM, improve glycemic level and reduce the occurrence of adverse pregnancy outcomes in women with GDM (18). Our study showed most participants lack exercise habit and foundation and the limited prior engagement in PA, shaped by both psychosocial and contextual factors was a primarily hinder of engagement exercise. Consistent with our study, evidence from the study in Ireland demonstrated that Most of the interviewed women did not enjoy exercise at any previous stage (25). A existing study have indicated that pre-pregnant exercise habits were a significant predictor of intent to initiate PA behavior (26). Furthermore, women with established pre-pregnancy exercise habits tend to exhibit higher levels of motivation and greater exercise intensity during pregnancy compared to those without such habits (27, 28).

Therefore, it may be necessary to cultivate healthy pre-pregnancy exercise habits to support optimal health-promoting behavior, and to encourage women to initiate and sustain safe physical activity before pregnancy (29, 30), which helps maintain sufficient exercise levels in the second and third trimester (31). Thus, intervening on these barriers will require the healthcare providers to develop a multi-professional involvement, theory-guided and evidence-based physical activity intervention, including knowledge education, exercise clinic visit and video, and group discussions with face-to-face and online blended sessions (32).

Another capability-related factor was physical discomforts during pregnancy. These discomforts such as fatigue, lower back pain, and tightness in the lower abdomen were common barriers reported by our participants. Furthermore, some participants in the study opined that they had insufficient coping strategies and limited knowledge for managing these discomforts, which often led to adopt inactive lifestyle and further decrease physical activity levels. These findings were also observed in other studies (33). Thus, professional support aimed at properly coping with physical discomforts was highlighted as critical.

Besides, some participants in our study who walked during commuting to work believed they had adequate physical activity and there was no need for them to exercise in leisure time. These findings indicate that insufficient knowledge and misconceptions regarding PA during pregnancy may contribute to ineffective or unsustainable activity patterns, underscoring the importance of providing individualized education and guidance tailored to address the knowledge gaps and misconceptions.

### 4.2 Opportunity-related factors

The COM-B component of opportunity was equally a key domain, including one enabling factor and two barriers. This study indicates that strong support from their families during pregnancy is a key facilitator of putting physical activity into action. The finding is consistent with a UK study, which found that women with GDM reported needing both emotional support from partners or close family and practical assistent with household responsibilities (including childcare) to allocate time for PA (34). Consequently, health management interventions for GDM women should fully activate the social support system, combining emotional and practical support to enhance self-management behaviors (35). Furthermore, the study showed that there was a mismatch between the demand for personalized exercise guidance among pregnant women with GDM and the guidance they receive from healthcare professionals. These results are consistent with the descriptions in existing literature healthcare professionals lack the knowledge and skills of pregnant exercise to provide appropriate guidance (36). To bridge this gap, healthcare providers need further training to enhance their knowledge and skills and GDM exercise guidelines should be constantly updated to incorporate Chinese cultural characteristics in order to offer the patients individualized care and scientific advice (37, 38).

Although prior studies suggested social support can significantly influence health-promoting behavior (39–41), the findings in our study was social support for GDM pregnant women very inadequate. The majority of participants in our study experienced limited peer support and community-based support, which affected exercise compliance to some extent. As such, effective interventions targeted GDM women’s partners family members and social networks during pregnancy (42), that may be complemented by peer support groups should be considered (43). In parallel, the establishment of online communities, such as WeChat groups dedicated to women with GDM, and the provision of antenatal education through multiple platforms can facilitate greater access to peer and professional support, create opportunities for physical activity, and ultimately improve exercise adherence (44).

### 4.3 Motivation-related factors

The COM-B component of motivation also needed to be taken seriously. Some participants in our study experience the fear of disease-related complications, particularly concerns regarding fetal health, which contributed to improve the compliance with physical activity. Existing research has demonstrated that fear of disease-related complications can act as a significant motivator for behavior modification in this population (45, 46). However, it was observed that some participants in this study exhibited low levels of risk perception. Thus, for women with limited risk perception, it is recommended that underlying causes be examined, followed by the provision of tailored guidance to facilitate active participation in disease risk management (47).

Some participants expressed that the concerns of safety of physical activity during pregnancy, especially the fear of harming their unborn baby was one of the barriers to participating in physical activity. Our study echoed the findings of Okafor et al., whose study demonstrated pregnant women felt concern about their safety and the baby during physical activity in pregnancy (48).

Other research has also found that pregnant women with GDM have a higher incidence of anxiety and depressive symptoms; additionally, compared with englycemic pregnant women, GDM pregnant wome with anxiety and depression were more likely to have adverse outcomes in terms of blood glucose during pregnancy, delivery mode, and maternal and infant outcomes (49).

Thus, expert advice and psychological support from healthcare providers during pregnancy were identified as crucial. GDM pregnant women with negative emotions require additional support: on the one hand, in the absence of contraindications, GDM patients should be encouraged to engage in physical activity and follow professional advice on prenatal physical activity, including guidance on the benefits of physical activity during pregnancy (50); On the other hand, cognitive-behavioral stress management training can help GDM patients reduce stress, and this intervention is suggested to be integrated into the standard treatment of GDM (51).

Among the motivation-related factors, the perceived benefits of physical activity identified in this study acts as an enabler. A subset of participants in our study recognized that engaging in physical activity could help them with blood glucose control, weight management, and improvement of physical discomfort. Consistent with our study, evidence from a Chinese study demonstrated that women with GDM who perceived the PA benefit were more willing to adhere to regular exercise (52). Therefore, it is crucial for healthcare professionals to emphasize the benefits of physical activity to pregnant women with GDM, even for those experiencing physical discomforts (33).

## 5 Limitations

Our study is limited by the fact that the sample was restricted to a single obstetrics clinic at a tertiary general hospital of China. Therefore, future researche with large scale sample sizes is needed to identify more comprehensive influencing factors of exercise compliance among women with GDM across China. Another potential limitation is the focus on a single population—women with GDM. Promoting sustained physical activity in this population requires coordinated efforts involving not only the women themselves but also family members, healthcare professionals, and community resources. Future research should adopt a multi-level, interdisciplinary approach to examine the complex factors influencing exercise adherence in this population. Such efforts will support the development of comprehensive, context-sensitive intervention programs aimed at promoting long-term engagement in physical activity during and beyond pregnancy.

## 6 Conclusion

In summary, this study adopted the COM-B model as its theoretical framework and employed a descriptive qualitative research design by conducting face-to-face interviews with 16 women diagnosed with GDM. Our findings revealed that limited engagement in pre-pregnancy and early pregnancy physical activity, challenges of physical discomfort, insufficient knowledge and skills regarding exercise during pregnancy, insufficient professional guidance, limited peer support and community-based support and negative emotions stemming from uncertainty about exercise safety during pregnancy were notable factors impacting compliance with exercise recommendations among women with GDM. These findings have clinical implications for healthcare professionals to design context-specific interventions to address these factors in order to promote compliance with exercise during pregnancy and physical activity level in this setting. A wider approach is also required, involving system-level interventions. The three-level linkage model of hospital-community-family may help improve the management of GDM, including increasing compliance with exercise recommendations, which requires significant organizational support to achieve.

## Data Availability

The original contributions presented in the study are included in the article/supplementary material, further inquiries can be directed to the corresponding author.
